# Decoding *BCR*::*ABL1* kinase domain mutations in chronic myeloid leukemia: Implications for tyrosine kinase inhibitor resistance in a South Asian population

**DOI:** 10.1016/j.htct.2026.106502

**Published:** 2026-07-28

**Authors:** Fatima Sharif, Rafia Mahmood, Maimoona Roghani, Sikandar Hayat Khan, Ibrahim Khan

**Affiliations:** a Department of Molecular Hematology, Armed Forces Institute of Pathology, Pakistan; b Department of Molecular Diagnostics, Armed Forces Institute of Pathology, Pakistan

**Keywords:** Chronic myeloid leukemia, Tyrosine kinase inhibitors, Philadelphia chromosome, Drug resistance

## Abstract

**Introduction:**

Treatment resistance has become increasingly common in patients with chronic myeloid leukemia. Lack of response to tyrosine kinase inhibitors is associated with mutations in the *BCR*::*ABL1* kinase domain. This study aims to report the frequency and types of *BCR*::*ABL1* kinase domain mutations in Pakistani chronic myeloid leukemia patients.

**Materials and methods:**

This prospective study was conducted from January to June 2025 at the Armed Forces Institute of Pathology in Pakistan. It included adult patients with chronic myeloid leukemia who were non-responsive to treatment based on molecular, hematological, and clinical criteria. Allele-specific real-time polymerase chain reaction was performed to detect four kinase domain mutations: T315I, E255V, E255K and Y253H. Data was analyzed using IBM SPSS v23.

**Results:**

The mean age of the 133 treatment-resistant chronic myeloid leukemia patients included in the study was 47.3 ± 13.6 years. The majority of cases (62.4%) were male and 79 (59.4%) patients were currently on imatinib therapy. The median *BCR*::*ABL1* level, as measured by quantitative polymerase chain reaction, was 30.73% (range: 1.17–100%) International Scale. *BCR*::*ABL1* kinase domain mutations were detected in 57 (42.9%) patients. E255K was the most common mutation detected in 45 (33.8%) cases, followed by E255V in 11 (8.3%), T315I in 1 (0.8%) and the Y253H mutation in 1 (0.8%) patient. The presence of kinase domain mutations was significantly associated with higher total leucocyte count (*p* = 0.002), while the E255K mutation was significantly associated with blast crisis (*p* = 0.048).

**Conclusion:**

This study revealed a high prevalence of *BCR*::*ABL1* kinase domain mutations in Pakistani chronic myeloid leukemia patients. E255K, the most frequently detected mutation, was significantly associated with blast crisis. These findings underscore the significance of molecular testing for personalized treatment.

## Introduction

Chronic myeloid leukemia (CML) is a hematological malignancy characterized by a reciprocal translocation between the *ABL1* gene on chromosome 9 and *BCR* gene on chromosome 22, resulting in the formation of the Philadelphia Chromosome [1]. This *BCR*::*ABL1* fusion gene codes for a protein that has constitutive tyrosine kinase activity, leading to uncontrolled cell proliferation [2]. According to the Global Burden of Disease database, the annual incidence of CML was approximately 35,380 cases in 2021, with a rising trend seen in countries with lower socio-demographic indexes [3]. Currently, CML accounts for approximately 15% of adult leukemias [4].

The advent of specific targeted therapy in the form of tyrosine kinase inhibitors (TKIs) represents a major breakthrough in the treatment of CML, drastically improving outcomes and life expectancy [5]. In 2001, imatinib mesylate was the first TKI to be approved by the Food and Drug Administration (FDA) for the management of newly diagnosed CML [6]. Based on guidelines from the European Leukemia Network (ELN) and National Comprehensive Cancer Network (NCCN), imatinib mesylate remains a standard first-generation TKI and a primary treatment option for treatment-naïve patients with CML [7,8].

However, in recent years, many studies have highlighted an emerging concern of resistance to TKI therapy among CML patients, with poor response to imatinib mesylate seen in approximately one-quarter of patients [9]. Resistance to TKI therapy is multifactorial, related to factors such as patient adherence to therapy, efficacy of drug metabolism and the presence of additional chromosomal abnormalities which confer a poor prognosis [10].

One of the most common causes of TKI resistance in CML is the presence of amino acid substitutions in the *BCR*::*ABL1* kinase domain (KD) [11]. With over 90 different KD mutations reported to date, these mutations cause TKI resistance by impairing binding of the drug to the BCR::ABL1 chimeric protein [12]. The identification of *BCR*::*ABL1* KD mutations has significant clinical implications - each mutation detected has a unique pattern of response to TKIs, therefore patient care should be tailored according to their specific KD mutation profile [13].

In the era of precision medicine, the detection of *BCR*::*ABL1* KD mutations is necessary to provide targeted therapy in CML. Therefore, the aim of this study is to report the frequency and types of *BCR*::*ABL1* KD mutations among Pakistani patients diagnosed with CML.

## Methods

This study was conducted from July 2024 to October 2025 at the Armed Forces Institute of Pathology, Pakistan. Adult patients diagnosed with CML who showed a poor response to the first-generation TKI imatinib were prospectively enrolled. Patients were included as confirmed cases of CML on the basis of the diagnostic criteria specified by the World Health Organization (WHO). Patients who failed to achieve a complete hematological response (CHR), complete cytogenetic response (CCR) or major molecular response (MMR) to treatment were included. Molecular response to frontline TKI treatment was defined based on criteria recommended by the European LeukemiaNet (ELN) guidelines [7].

• *BCR*::*ABL1* transcript levels >10% at three months after initiating TKI therapy.
• *BCR*::*ABL1* transcript levels >1% at six months after initiating TKI therapy.
• *BCR*::*ABL1* transcript levels >0.1% at 12 months after initiating TKI therapy.
• Loss of major molecular response or detection of additional cytogenetic abnormalities at any time during treatment.

Patients who were started on imatinib at the time of diagnosis and subsequently empirically switched to second line treatment due to poor tolerance and excessive side effects were also included.

For the detection of *BCR*::*ABL1* KD mutations, a 3-mL peripheral blood sample was collected in an ethylenediaminetetraacetic acid (EDTA) anticoagulant tube. From this sample, RNA was extracted using the Thermo Fisher RNA Extraction kit, which works on the principle of silica column-based extraction of total RNA. After RNA extraction, complementary DNA (cDNA) was prepared. The quantity and quality of cDNA was assessed using a Microvolume Spectrophotometer (BioTek Epoch), with target cDNA quantity greater than 10 ng/µL and an A260/A280 ratio of 1.6:2.0 as acceptable DNA purity.

To identify mutations within the *BCR::ABL1* KD, allele-specific real-time PCR was carried out using cDNA. Primer sequences for four common KD mutations, namely T315I, E255V, E255K and Y253H, were derived from a previous study by Wang et al. [14]. This study tested for these four mutations due to their significant therapeutic implications and high prevalence reported in previous studies. Initially, the results of twenty samples were further confirmed by Sanger sequencing. Subsequently, aliquots of patient cDNA that tested positive for KD mutations were prepared and stored at −20 °C to be used as positive controls in each run. Normal samples were used as negative controls.

A structured data collection form was designed to collect information regarding patient demographics, laboratory parameters, history of TKI therapy and the presence of *BCR*::*ABL1* KD mutations. Data was entered and analyzed using the IBM Statistical Package for Social Sciences (SPSS) Software v23.

This study was initiated after receiving ethical approval from the Institutional Review Board (IRB) of the Armed Forces Institute of Pathology. Patient confidentiality was maintained throughout the study, and no external funding was received.

## Results

A total of 780 diagnosed cases of CML presented to our center during the study period. Among them, 133 patients with poor response to therapy were included in this research. The mean age of the study participants was 47.3 ± 13.6 years and the majority (*n* = 83; 62.4%) were male. The median time since diagnosis was 12 months (range: 1–246 months). Most of the patients (*n* = 108; 81.2%) were in the chronic phase of CML at the time of testing for KD mutations, while 12 (0.9%) cases had progressed to blast crises.

The median quantitative PCR for the *BCR*::*ABL1* transcript level was 30.73% (range: 1.17 – 100%) International Scale. The primary indication for *BCR::ABL1* KD mutational analysis was an inadequate molecular response to TKI treatment (52%; *n* = 69). Additional triggers for testing included a failure to achieve hematological response (13.5%), progression to high-risk CML or blast crisis (12%), and clinical suspicion (22.5%).

Most of the patients (*n* = 79; 59.4%) were on imatinib therapy at the time of KD mutation analysis, while the remaining had been empirically switched to second or third generation TKIs based on clinical suspicion of TKI resistance. Of these, 23 (17.3%) were receiving nilotinib, five (3.8%) dasatinib, two (1.5%) ponatinib and one (0.8%) patient was on asciminib therapy. One patient (0.8%) had undergone hematopoietic stem cell transplantation due to failure of multiple therapies.

**Figure 1:**
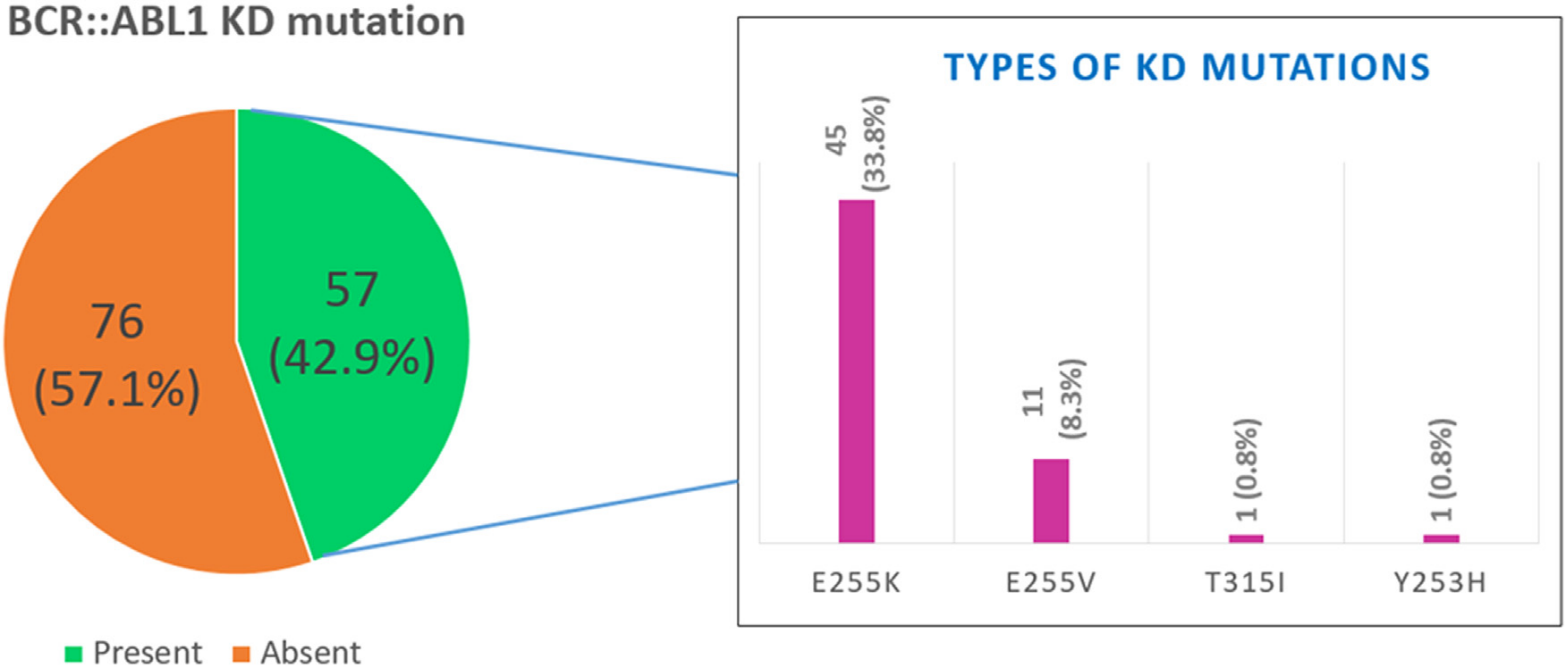
Frequency and types of *BCR*::*ABL1* kinase domain mutations in the study population.

Of the 133 patients tested, 57 (42.9%) showed the presence of *BCR*::*ABL1* KD mutations. E255K was the most frequently detected mutation in 45 (33.8%) patients. Furthermore, E255K was the only KD mutation identified in patients with progression to blast crisis. Figure 1 shows the proportions of *BCR*::*ABL1* KD mutations detected in the study population.

Table 1 summarizes the clinicopathological characteristics of the study population based on their *BCR*::*ABL1* KD mutation status.

**Table 1:** Clinicopathological characteristics of the study population according to presence of BCR::ABL1 kinase domain mutations.

	*BCR*::*ABL1* KD Mutation		p-value
	Present (*n* = 57)	Absent (*n* = 76)	
Gender			
Male - n (%)	37 (65)	46 (60.5)	0.823
Age (years) - (Mean ± SD)	48.2 ± 14.1	46.6 ± 13.2	0.523
Duration of CML (months) - Median (range)	13.5 (1 – 246)	12.0 (1 – 192)	0.425
*BCR*::*ABL1* qPCR (% IS) - Median (range)	30.73 (1.17 – 100.00)	30.64 (1.53 – 100)	0.553
Hemoglobin (g/dL) - (Mean ± SD)	11.49 ± 2.47	11.54 ± 2.24	0.906
Total leucocyte count (× 10⁹/L) - Median (range)	**10.2 (3.6 – 237.0)**	**7.6 (0.9 – 330.0)**	**0.002**
Platelet count (× 10⁹/L) - Median (range)	253 (21 – 1173)	215 (11 – 1577)	0.608
TKI used - n (%)			
Imatinib	33 (57.9)	46 (60.5)	0.350
Other	16 (28.1)	15 (19.7)	
Blast crisis - n (%)	7 (12.3)	5 (6.6)	0.240

CML: Chronic myeloid leukemia; TKI: Tyrosine kinase inhibitors.

The presence of *BCR*::*ABL1* KD mutations was significantly associated with a higher median total leucocyte count (*p* = 0.002) (Table 1).

Further analyses identified factors associated with the presence of E255K, the most frequent type of *BCR*::*ABL1* KD mutation in this study population (Table 2). The E255K mutation was significantly associated with higher TLC (*p* = 0.001) and progression to blast crisis (*p* = 0.048).

**Table 2:** Clinicopathological characteristics associated with presence of E255K mutation.

	E255K Mutation		p-value
	Present (*n* = 45)	Absent (*n* = 88)	
Gender			
Male - n (%)	28 (62.2)	55 (62.5)	0.779
Age (years) - (Mean ± SD)	47 ± 14.8	47.4 ± 13.0	0.866
Duration of CML (months) - Median (range)	15 (1 – 246)	10.5 (1 – 192)	0.247
*BCR*::*ABL1* qPCR (%IS) - Median (range)	40.18 (1.17 – 100)	30.08 (1.53 – 100)	0.226
Hemoglobin (g/dL) - (Mean ± SD)	11.16 ± 2.32	11.70 ± 2.33	0.244
Total leucocyte count (× 10⁹/L) - Median (range)	**12.8 (3.6 – 237.0)**	**7.7 (0.9 – 330.0)**	**0.001**
Platelet count (× 10⁹/L) - Median (range)	237 (21– 1173)	215 (11 – 1577)	0.738
TKI used - n (%)			
Imatinib	25 (55.6)	54 (61.4)	0.182
Other	14 (31.1)	17 (19.3)	
Blast crisis - n (%)	**7 (15.6)**	**5 (5.7)**	**0.048**

CML: Chronic myeloid leukemia; TKI: Tyrosine kinase inhibitors.

## Discussion

This study assessed the presence of four common *BCR*::*ABL1* KD mutations in Pakistani CML patients. Based on existing reports, mutations affecting the *ABL* KD have emerged as the predominant driver of TKI resistance in CML [15]. *BCR*::*ABL1* KD mutation analysis is recommended by both the National Comprehensive Cancer Network (NCCN) and European LeukemiaNet (ELN) guidelines for patients who are not achieving an adequate response to TKI therapy [7,16].

The overall frequency of *BCR*::*ABL1* KD mutations was 42.9%. There is a wide variation in the literature regarding the frequency of *BCR*::*ABL1* KD mutations in different patient populations (Table 3) with the reported prevalence ranging from 13%−71%. The prevalence depends on many factors such as the patient demographics, method of detection, TKIs used, phase of disease and other clinicopathological characteristics [17, 18, 19, 20, 21, 22].

**Table 3:** Summary of previous studies reporting the frequency of BCR::ABL1 kinase domain (KD) mutations.

Author	Year	Country	n	Frequency of KD mutations	Most common mutations
Mahboobeh S et al. [17]	2021	Iran	50	13 (26.0%)	E255K (20%)
Hasanova et al. [18]	2023	Azerbaijan	163	22 (13.4%)	T315I (5%)
Elias et al. [19]	2014	Malaysia	125	28 (22.4%)	T315I (7.2%) E255K (3.2%)
Datta et al. [20]	2024	India	108	62 (71%)	T315I (46%)
Kizilors et al. [21]	2019	UK	121	25 (21%)	F317L (20%) M244V (14%)
Jabbour et al. [22]	2009	USA	169	86 (51%)	G250E (16%)

This study found a high prevalence of the E255K mutation, seen in approximately one-third of the study population. The E255K point mutation at codon 255 in the *ABL1* KD results in a single amino acid substitution of glutamic acid by lysine in the subsequent protein sequence. This mutation, located in the phosphate binding loop (P-loop) of the *ABL* KD, confers resistance to imatinib and is associated with a poor prognosis [23]. According to the latest ELN guidelines published in 2025, the recommended TKI to use in patients harboring this mutation is ponatinib, while dasatinib or asciminib may also be used [7].

This finding aligns with a study from Iran, which also identified E255K as the most common *ABL1* KD mutation in 20% of patients with TKI resistance [17]. However, while E255K was the most frequently encountered mutation in the present cohort, many studies have identified the T315I mutation as the most common variant within the *BCR::ABL1* KD [18, 19, 20]. The T315I mutation comprises a single amino acid substitution of threonine by isoleucine at codon 315 of the *ABL* KD. It is considered a ‘gatekeeper’ mutation because its location is at the position where TKIs form hydrogen bonds, which enables stable drug binding [24].

In this study, a higher total leucocyte count was significantly associated with presence of *BCR*::*ABL1* KD mutations. Furthermore, the presence of the E255K mutation was significantly associated with blast crisis in CML patients. A study from China reported several factors that were associated with the development of *BCR*::*ABL1* KD mutations including young age, male gender and advanced phase of disease [25]. However, these variables did not reach statistical significance in the current cohort. Such discrepancies may be attributed to population diversity; nonetheless, methodological factors, specifically testing techniques and follow-up strategies, must also be considered.

Table 3 summarizes the results of previous studies that have reported the frequency and types of *BCR*::*ABL1* KD mutations in different patient populations.

Allele-specific RT-PCR was used for detecting these mutations; this is a cost-effective strategy that is feasible in low-resource settings. A recent study which compared the diagnostic utility of RT-PCR with Next Generation Sequencing (NGS) found that PCR gave highly sensitive and reliable results, detecting mutations at an estimated variant allele frequency as low as 0.01% [26]. Despite its high sensitivity, this methodology is restricted to a predefined panel of mutations. Unlike Sanger sequencing or NGS, which can screen the entire kinase domain, allele-specific PCR is limited by the specificities of the allele-specific primers employed [27].

The findings of this study highlight the importance of *BCR*::*ABL1* KD mutation testing and provide the foundation for personalized treatment strategies. However, future studies should assess the presence of these mutations in a large sample employing NGS, which would allow the detection of the full spectrum of *BCR*::*ABL1* KD mutations.

## Conclusion

This study identified a high frequency of *BCR::ABL1* KD mutations within this CML population; notably, E255K was the most prevalent variant and was significantly associated with blast crisis. This report of *BCR*::*ABL1* KD mutations in Pakistani CML patients is essential to provide targeted treatment. These findings should be validated in a larger cohort using NGS to capture a broader spectrum of *BCR::ABL1* KD mutations.

## Funding

This research did not receive any specific grant from funding agencies in the public, commercial, or not-for-profit sectors.

## Conflicts of interest

None of the authors have any conflicts of interest to declare.

## Data Availability

The data that support the findings of this study are available from the corresponding author upon reasonable request.
